# Structural, radiation shielding, thermal and dynamic mechanical analysis for waste rubber/EPDM rubber composite loaded with Fe_2_O_3_ for green environment

**DOI:** 10.1038/s41598-024-62308-4

**Published:** 2024-05-30

**Authors:** M. M. AbdelKader, M. T. Abou-Laila, M. S. S. El-Deeb, Eman O. Taha, A. S. El-Deeb

**Affiliations:** 1 Housing and Building National Research Center, Building Physics Institute, Giza, Egypt; 2 Radiation Safety Department, Nuclear and Radiological Safety Research Center, Egyptian Atomic Energy Authority, Cairo, Egypt; 3 Manufacture Engineering and Production Technology Department, Modern Academy for Engineering and Technology, Cairo, Egypt; 4 Petroleum Applications Department, Egyptian Petroleum Research Institute (EPRI), Cairo, Egypt

**Keywords:** Waste rubber, EPDM, Radiation shielding, DMA, Gamma attenuation, Materials science, Applied physics

## Abstract

Increasing waste rubber recycling produces a specious range of products for many valuable applications. Waste Rubber/EPDM composite with different concentrations was prepared. Infrared spectroscopy (FTIR) is used to identify the chemical composition. A water absorption test, Dynamic mechanical analysis (DMA), and Thermal Gravimetric Analysis (TGA) were performed. The (75/25) WR/EPDM rubber composite exhibited the best behavior with the highest mechanical performance. Fe_2_O_3_ was added to (75/25) WR/EPDM rubber composite. Water absorption, FTIR, TGA, and DMA were investigated. The composite performance was improved with increasing Fe_2_O_3_ content. The linear attenuation coefficients (μ) were also measured as a function of the concentrations of Fe_2_O_3_ for γ-ray energy 662 keV by using 137Cs point source; the radiation shielding can be denoted by numbers of parameters like mass attenuation coefficient (μm), half value layer (HVL), Tenth value layer TVL and radiation protection efficiency (RPE%), radiation protection efficiency increased as Fe_2_O_3_ increased.

## Introduction

Rapid population growth, along with increasing productivity and consumption has aggravated waste production. Many researchers were directed to the use of waste materials for more ecological environment^1–3^. The accumulation of waste creates several problems. These problems are worse when waste accumulates at incorrect sites^4^. The poor solid waste management problems have become one of major concerns for a number of environmental events. It is an important element to consider, the preservation of individual’s health and ensuring environment safeguard. Tons of forsaken tires pose big environmental dilemma due to improper disposal method, so recycling prevents those junked tires to cast down in landfills, lakes, seas and along the side of the roads. Many useful products can be made from recycled tires with properties better than their normal alternatives such as rubberized asphalt that are sturdier and provides more sliding resistance than normal paving materials due to the known durability and skid properties of rubber. Flooring, playground turf and railroad ties are also products to recycled rubber. Using some metals or transition metals with polymer loaded with wastes can produce lightweight low cost shielding materials^5,6^.

By utilizing high ratios of waste rubber- rubber composite, eco-friendly, low cost, innovative waterproofing membranes were produced with qualities comparable to their contemporary market substitutes^7^. Mechano-chemical reclamation to waste EPDM rubber by using disulfide oil (DSO) waste produced by gas refinery (as a chemical agent) under specified conditions was executed, the unexpected consequence was a 73% decrease in crosslinking density of reclaimed EPDM, blending up to 40% reclaimed EPDM rubber (RR) with virgin EPDM rubber improves its mechanical properties while has no effect on optimum curing time, curing rate, or scorch time which is profitable towards managing the quandaries of waste rubber eradication and disulfide oil (DSO)^8^. In terms of health, outdoor air quality, and material costs, it is beneficial to consider the possibility of using tire material in the building sector due to its acceptable capabilities, such as outstanding resistance to weather circumstances, moisture, and temperature. Numerous studies have demonstrated that reusing tires in building and construction projects greatly improves their environmental friendliness. Tire recycling also provides solutions to environmental problems while cutting expenses and lowering the need for renewable resources ^9,10^. The examination of the properties of concrete with shredded tire rubber demonstrated that acceptable elastomeric concrete blends may be created from scrap tire rubber. Though the weakened compressive property of concrete mixed with rubber particles may restrict its applicability for particular structures. However, rubberized concrete also offers some advantages, including lower density, increased impact and toughness resistance, improved ductility, and increased sound insulation^11^.

The prevalent use of X-ray and gamma-ray sources in medicinal and industrial applications necessitates a thorough understanding of their potential risks. These radiations possess the ability to ionize matter through intricate mechanisms, leading to the release of secondary charged particles. Notable processes include the photoelectric effect, incoherent (Compton) scattering, coherent (Rayleigh) scattering, and, for photon energies exceeding 1022 keV, nuclear-field pair production and atomic-field production. Due to the inherent risks posed by these radiations to individuals, environments, and materials undergoing degradation, the imperative to enhance radiation shielding materials becomes evident. Traditional options like lead and mercury, while effective, present inconveniences due to their lack of flexibility, high density, toxicity, and the generation of bremsstrahlung during electron interaction^12^. As applications expand, there arises a critical need for cost-effective and lightweight shielding material^13^. In response to this demand, polymer composites filled with metals or metal oxides have emerged as favorable alternatives. This is particularly relevant in nuclear medicine, where the weight of lead shields proves cumbersome for personnel. Metal-filled polymers offer a lightweight solution suitable for shielding both individuals and materials. These composites find applications in medical equipment and instruments generating gamma rays or X-rays, safeguarding occupants, patients, and electronic components from tube leakage or backscattering^14^. The versatility of these polymer-metal composites extends to applications such as X-ray tube insulation, radioisotope housings, and wall coverings in nuclear medicine treatment and diagnostic rooms. The key lies in utilizing polymer-metal composites with optimal mechanical and physical properties to create flexible and lightweight gamma-ray shielding materials. In this context, rubber-based radiation shielding materials have emerged as superior alternatives to lead-based shields^15,16^.

The Waste/EPDM samples, exhibiting commendable characteristics, led to the incorporation of ferric oxide into the sample with the highest waste concentration. This strategic enhancement aims at addressing the specific requirements of medical fields, especially in nuclear medicine departments where most of the common pharmaceutical radionuclides do not exceeds 640 keV, and in X-ray diagnostic rooms the energy range (10–150keV) where low energy is utilized. The resulting lightweight material serves as an effective wall lining, reducing the reliance on lead bricks or sheets for shielding. This innovation not only maintains the necessary protective measures but also mitigates the emittance of backscattered radiation. In conclusion, polymer-metal composites represent a cutting-edge solution, balancing efficiency, cost-effectiveness, and flexibility, advancing the field of gamma-ray shielding materials.

## Experimental

### Materials and method

The materials employed in this study were sourced from the Alexandria factory for tire manufacture, Egypt, comprising the following components: EPDM (Ethylene Propylene Diene Monomer) with ethylene content of 54% and density of 0.86 g cm^−3^ was supplied by Arlanxeo performance elastomers, Netherlands. Zinc oxide (ZnO) utilized as an activator with a specific gravity ranging from 5.55 to 5.61, Stearic acid with a melting point of 67–69 °C and a specific gravity of 0.838, Tetramethyl thiuran disulfide (TMTD) serving as an accelerator with a melting point of 148.5 °C and a specific gravity between 1.29 and 1.31, Antioxidant N-isopropyl N0-cyclohexyl paraphenylene diamine (IPPD) with a density of 1.17 g/cm^3^, Sulfur having a specific gravity range of 2.04–2.06, and Naphthenic oil characterized by a viscosity of 80–90 poise at 100 °C and a specific gravity between 0.94 and 0.96. The waste rubber powder obtained from recycled tires is produced by removing the metallic wires from old tires and then crushing them. To determine the elemental composition of the waste rubber tires, X-ray fluorescence analysis (XRF) was conducted using Multi-purpose Bench top Sequential Wavelength Dispersive X-Ray Florescence spectrometer, model supermini 200 (Japan). The data represented in Table 1.

**Table 1:** XRF of waste rubber powder.

Constituents wt%	Sample
MgO	0.260
Al_2_O_3_	0.270
SiO_2_	5.77
P_2_O_5_	0.114
SO_3_	8.19
Cl	0.084
K_2_O	0.237
CaO	1.27
TiO_2_	0.152
MnO	0.055
Fe_2_O_3_^tot^	3.36
Zn	8.54
L.O.I	70.80
Traces ppm	
Br	1960
Cu	6280
Co	777

Experimental samples for the tests were formulated according to the recipe outlined in Table 2. The mixing and blending processes were executed using two-roll mills, involving a two-step procedure. The initial step involved creating a master batch containing refined oil, activators, antioxidants, antiozonants, reinforcing agents, waste rubber, and Fe_2_O_3_. Subsequently, the final batch was prepared by combining the original master batch with curing agents (Sulfur) and accelerators, the latter added recently to prevent pre-vulcanization resulting from elevated temperatures. Vulcanization of the rubber compounds took place at a temperature of 150 °C and a pressure of 150 bar for a duration of 15 min in an electrically heated uniaxial press. Diverse moldings were employed to fabricate samples tailored to specific test requirements. This systematic procedure ensured the consistent and controlled preparation of rubber composites for subsequent experimental analyses.

**Table 2:** Shows the composition of prepared samples.

Ingredients (wt%)	Samples						
	S1	S2	S3	S4			
EPDM	100	75	50	25			
Waste Rubber	0	25	50	75			
				S4	S4F1	S4F2	S4F3
Fe_2_O_3_	–	–	–	0	50	100	150
Stearic acid	2	2	2	2			
Zinc oxide	5	5	5	5			
Processing Oil	10	10	10	10			
TMTD	2	2	2	2			
IPPD (4020)	1	1	1	1			
Sulphur	2.5	2.5	2.5	2.5			

**Figure 1:**
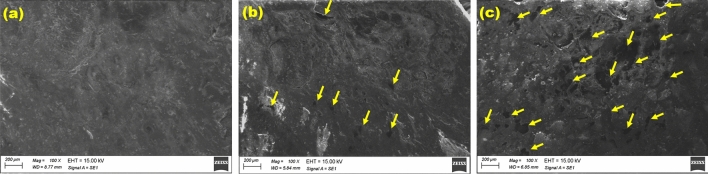
Show SEM images of the surface view for samples (**a**) S4, (**b**), S4F1 and (**c**) S4F3, respectively.

The miscibility of fabricated samples was examined using the Scanning Electron Microscope (SEM)(Sigma 300 VP, Carl Zeiss). Figure 1a–c show SEM images of the surface view of samples, S4, S4F1 and S4F3, respectively. The SEM images demonstrate that the composite materials are homogeneously blended, with no visible signs of phase separation. The images highlight effective miscibility among the rubber matrix, waste tire powder, and Fe_2_O_3_.

Notably, As the concentration of Fe_2_O_3_ increases, pore formation occurs inside the matrix. Increased Fe_2_O_3_ concentration leads to the proliferation of these pores (yellow arrows in the images). The high molecular weight of Fe_2_O_3_ (159.69 g mol^−1^) may enhance pore development in composite structures.

### Characterizations

In adherence to ASTM D792 standards^17^, the specific gravity of samples was determined. These samples, measuring (20 × 20 × 3) mm, underwent rigorous testing to unravel their water absorption characteristics, following ASTM D570 standards^18,19^. Samples, with dimensions (100 × 100 × 2) mm, were subjected to a 24 h immersion in a water bath. The weight before and after soaking was meticulously recorded.

Dynamic Mechanical Analysis (DMA) was conducted on a Triton Instruments, employing the tension mode at a frequency of 1 Hz. The temperature range spanned from − 75 to 23 °C, and the scanning rate was maintained at 3 °C/min. Surface chemical changes were scrutinized using an Alpha Bruker platinum Attenuated Total Reflection-Fourier Transform Infrared Spectroscope (ATR-FTIR), featuring a wavenumber range of 600–4000 cm^−1^. Thermogravimetric analysis (TGA) was conducted on a Mettler-Toledo (TA-TGA). The sample was heated to temperatures ranging from 36 to 700 °C in a controlled nitrogen gas atmosphere with heating rate was 10 °C/min.

**Figure 2:**
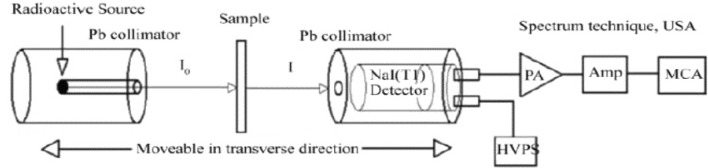
The experimental configuration for the gamma-ray spectrometer*.*

The investigation delved into the linear and mass attenuation coefficients (μ and μm, respectively) of the composite. These coefficients quantify the likelihood of photon interactions with shielding medium and their probability per unit distance, and area per mass transversed through materials. Utilizing a gamma-ray spectrometer system with a NaI (Tl) scintillation detector (Oxford model) with 3″ × 3″ amplifier and multi-channel analyzer 16 k channels with narrow beam geometry, the linear attenuation coefficient was measured. Equation (1) facilitated the calculation of the density (ρ) of the polymer matrix, a crucial factor in subsequent computations^12^.

1 $$\rho =\frac{M}{V}$$

where M represents the mass (g) and V represents the volume (cm^3^) of each sample. The γ-ray spectrometer system, depicted in Fig. 2, was instrumental in measuring μ (cm^−1^). The experimental μ values, coupled with the density of the polymer matrix, enabled the calculation of *μ*_m_ (cm^2^/g) for all composite samples, specifically for gamma-rays with energy 662 keV emitted by a ^137^Cs point source, which located 7cm from the detector.

The Beer-Lambert's law guided the determination of μ, where I represents the radiation intensity passing through the absorbent material of thickness x, and Io is the radiation intensity without the absorbent medium^20^.

2 $$I = I_{0} e^{(-\mu x)}$$

To assess the penetration capacity of radiations and materials, half value layer (HVL) and tenth value layer (TVL) were quantified using the equations^21^:

3 $$\text{HVL}=\frac{ ln2}{\mu }, \text{TVL}=\frac{ ln10}{\mu }$$

These values provide critical insights into radiation shielding calculations. Where HVL is the thickness of the absorber required to reduce incident radiation intensity to one-half of its initial value and TVL to one-tenth of its initial value.

Additionally, the Radiation Protection Efficiency (RPE%) which evaluation the ability of composite in attenuating gamma rays was evaluated using the equation^9^:

4 $$RPE\%= \left(1- \frac{I}{{I}_{0}}\right)\times 100 \%$$

Finally, the average distance between two subsequent interactions, denoted by the photon mean free path or relaxation length (λ), was determined^22^:

5 $${\varvec{\lambda}}=\frac{1}{{\varvec{\mu}}}$$

## Results and discussion

### Specific Gravity

**Figure 3:**
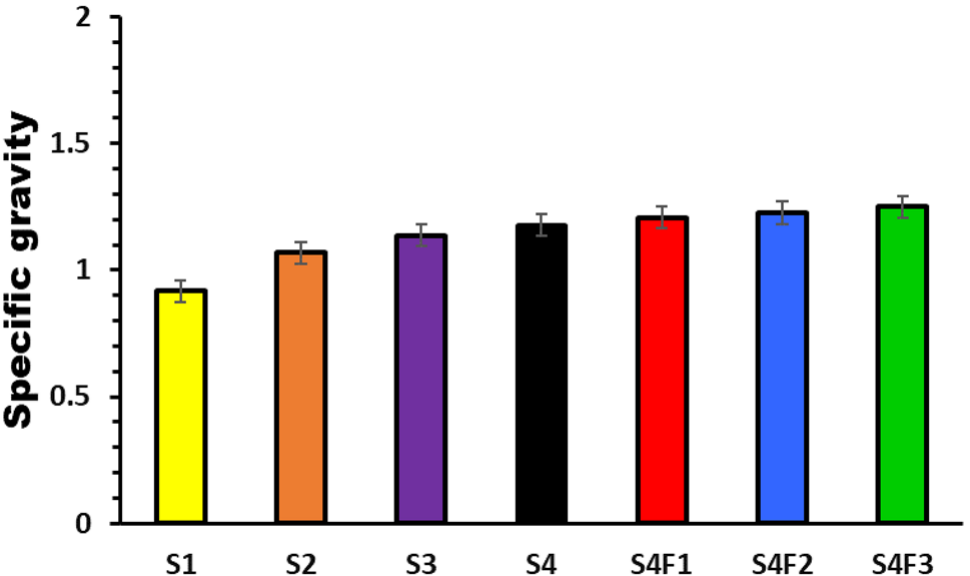
Specific gravity for various waste rubber concentrations and Fe_2_O_3_ concentrations.

Specific gravity is significant quality index for most industrial products. The presence of pores within a sample evidenced by a decrease in specific gravity values, which is undesirable in many applications. The specific gravity (ρ) was estimated using Eq. (6)^23,24^ via various concentrations of waste rubber (WR) and ferric oxide (Fe_2_O_3_) applied to a composite (75/25 WR/EPDM) Fig. 3. An increase in specific gravity with waste rubber, also by addition of Fe_2_O_3_ specific gravity increases, which is an adequate sign that the samples were correctly prepared.

### Water absorption measurements

The capacity of material to absorb moisture from surrounding environment is known as water/moisture absorption. This moisture can affect the glass transition temperature and strength of material which also can lead to irreversible degradation of material structure as it can cause dimensional and mass changes (swelling) also the performance of the material during processing can be studied. In addition to the presence of moisture in the structure of material influences the thermal insulation and dielectric properties also it is one of the factors that can cause material aging^25^. Water absorption was calculated according to ASTM D 570 after 24h immersion according to equation

6 $$\text{Water absorption coefficient \% }= \frac{\text{wet sample}-\text{conditioned sample}}{\text{conditioned sample}}$$

**Figure 4:**
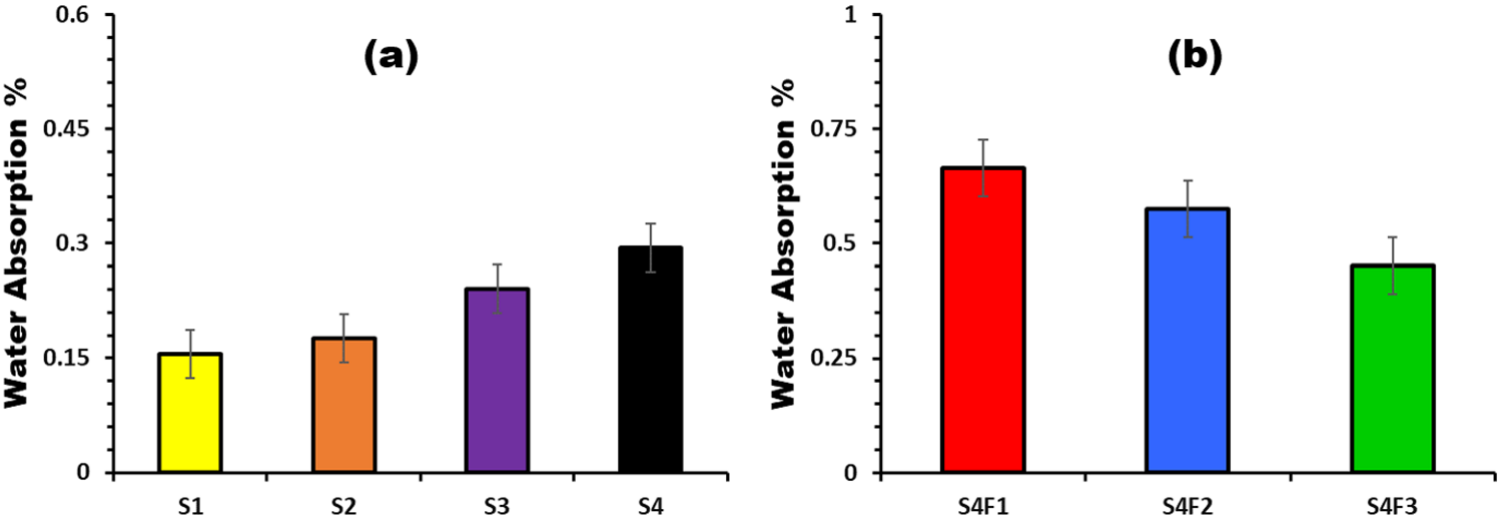
Water absorption % for (**a**) different waste rubber concentrations and (**b**) Fe_2_O_3_ concentrations.

Figure 4 shows the water absorption coefficient of samples via the various waste rubber concentrations and via various Fe_2_O_3_ concentrations. An increase in water absorption coefficient with the increase of waste material concentrations, while a decrease in water absorption coefficient with increasing Fe_2_O_3_ content. All samples are in the acceptable range of building applications^11^. It is possible to explain the observed increase in water absorption percentage to the high molecular weight of Fe_2_O_3_ (159.69 g mol^−1^). This property may facilitate the formation of pores within the sample and it is well proved by SEM images (Fig. 1).

### ATR–FTIR measurements

**Figure 5:**
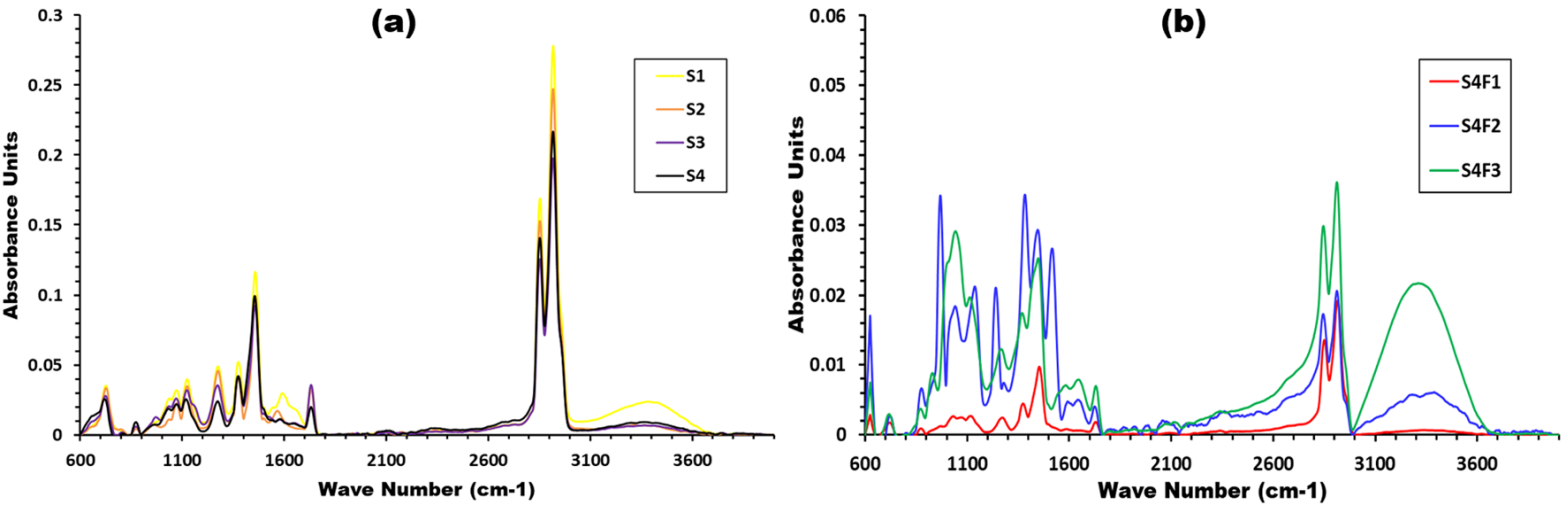
The ATR–FTIR spectra of (**a**) different waste rubber concentrations and (**b**) Fe_2_O_3_ concentrations.

Fourier transform infrared spectroscopy (ATR-FTIR) is a swift, frugal, simple and non-destructive technique for confirming pure compounds identity that based upon functional group identification with molecule, where this groups vibrates through stretching or bending in assorted ways. Figure 5a exhibits the ATR–FTIR spectra of the prepared samples with different waste rubber concentrations. Absorption arising from C–H stretching occurs in the general region of 3370–2840 cm^−1^, where the C–H symmetric and asymmetric stretching vibration are at around 2916 cm^−1^ and 2850 cm^−1^, respectively. Moreover, absorption due to the bending vibrations of the C–H bonds of methyl and methylene group occurs around 1460, 1375, and 720 cm^−1^ ^26^ absorption due to the skeletal complex shape of vibrations occurs in region 1305–1000 cm^−1^. Also, absorption due to the vibrations of dienes part of macromolecule chain are observed at 1600, 1275, 872 and 805 cm^−1^ ^27^. Figure 5b exhibits the ATR–FTIR spectra of the prepared samples with different Fe_2_O_3_ concentrations. This curve exhibits a vibration at 630 cm^−1^ for all sample this peak may be due the bending vibration of iron-oxygen (Fe–O) bond^27,28^. All functional groups and vibration types are listed in Table 3.

**Table 3:** ATR-FTIR spectrum of a sample in the range of 600 to 4000 cm^-1^.

Functional group	Vibration type	Spectral region in cm^-1^
C–H	Stretching	3370-2840 ^26^
C–H	Symmetric stretching	2916 ^26^
C–H	Asymmetric stretching	2850 ^26^
C–H of methyl and methylene group	Bending	1460-1375-720 ^26^
Skeletal complex shape	Absorption due to vibration	1305-1000 ^27^
C_n_H_2n-2_ dienes	Absorption due to vibration	1600-1275-872–805 ^27^
Fe–O	Bending	630 ^26–28^

### Thermal properties

**Figure 6:**
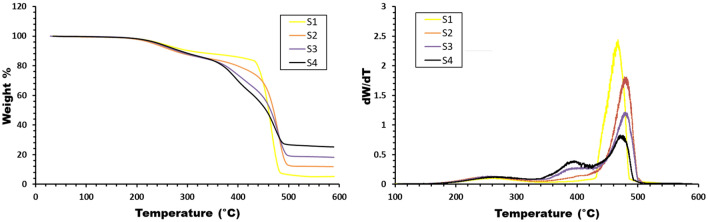
TG–DTG graphs of weight (%) and derivative weight (%) versus temperature for samples with different waste rubber concentrations.

**Figure 7:**
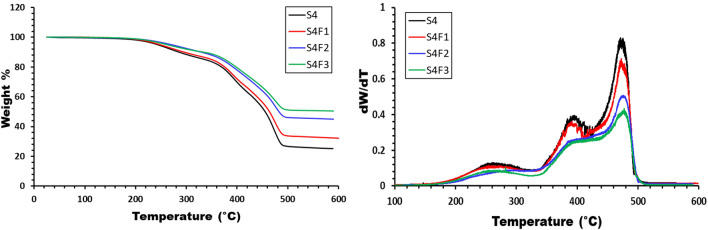
TG–DTG graphs of weight (%) and derivative weight (%) versus temperature for samples with different Fe_2_O_3_ concentrations.

The investigation of material thermal stability is effectively conducted through thermogravimetric analysis (TGA), wherein changes or losses in weight are measured with increasing temperature. This weight variation can arise from processes such as decomposition, evaporation, combustion, or oxidation. Figures 6 and 7 present TGA and derivative thermogravimetric (DTG) graphs, respectively, depicting the percentage weight and derivative weight as functions of temperature. These graphs encompass different concentrations of waste rubber and Fe_2_O_3_, employing a heating rate of 15 °C/min within the temperature range of 30–600 °C.

For pure EPDM, the curve reveals a singular decomposition step, with the peak decomposition temperature occurring at 460°C. However, the introduction of waste rubber and Fe_2_O_3_ prompts a three-step decomposition process. The initial decomposition, observed between 180 and 280°C, signifies the onset of material disintegration, possibly attributed to the evaporation of physically weak bonds and chemically strong-bound water within the sample^29^.

The second decomposition, transpiring between 350 and 420°C, may be attributed to the decomposition of hydrogen bonds within the matrix. The third decomposition, occurring between 450 and 490 °C, suggests the cleavage of the C–C backbone (carbonation) of the polymer itself^30^.

An interesting observation emerges with the incorporation of waste rubber, manifesting as a discernible shift in thermal decomposition, indicating an enhancement in the thermal stability of EPDM rubber. Additionally, a marginal improvement in thermal stability is noted with the incremental addition of Fe_2_O_3_. These findings underscore the impact of compositional variations on the thermal behavior of EPDM-based composites, offering valuable insights into the tailored manipulation of thermal properties for diverse applications in polymer science.

### Dynamic mechanical properties

**Figure 8:**
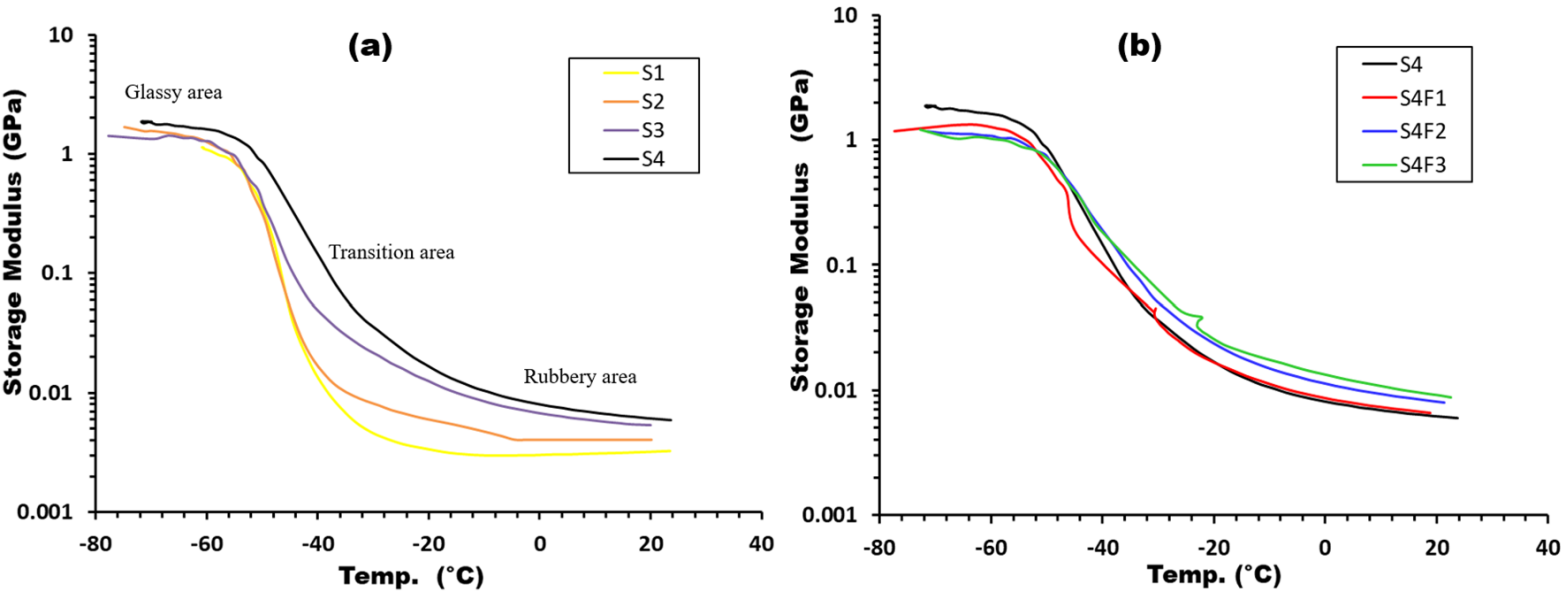
exhibits storage modulus vs. temperature for samples with (**a**) different waste rubber concentrations and (**b**) Fe_2_O_3_ concentrations.

In the exploration of dynamic mechanical properties, a dynamic analysis was conducted at a frequency of 1 Hz to assess the mechanical characteristics of formulated samples. Figure 8a, b vividly portray the storage behavior of WR/EDPM rubber composites with varying concentrations of waste rubber and (75/25) WR/EDPM rubber with different concentrations of ferric oxide at diverse temperatures. The dynamic mechanical response revealed three distinct regions as the temperature increased. Firstly, the “glassy area” displayed a high modulus, attributed to restricted movement of rubber chains. Subsequently, the "transition area" exhibited a modulus decline, signifying the presence of glass transition temperatures. Finally, the "rubbery area" featured low modulus values, indicating heightened mobility of rubber chains and energy dissipation^31,32^.

Variations in waste rubber concentration directly influenced storage modulus values. Figure 8a illustrates that the pure EPDM sample (S1) had the lowest storage modulus, with an incremental rise as waste rubber content increased until reaching a peak for sample S4. At 20 °C, storage modulus values for samples S1, S2, S3, and S4 were 3.2 MPa, 4.1 MPa, 5.3 MPa, and 6.2 MPa, respectively, denoting a substantial 93% increase for sample S4 compared to S1. The addition of Fe_2_O_3_ further elevated storage modulus, indicating enhanced rubber matrix stiffness (Fig. 8b). The detailed values of the storage modulus can be found in Table 4.

**Table 4:** DMA data for prepared samples.

Samples	Storage modulus (MPa) at room Temp. & Freq. 1 Hz	T_g_ (°C)	Height of tan δ peak	FWHM	E_a_ (kJ/mol)
S1	3.2	− 45.5	1.307	13	462.7
S2	4.1	− 43.9	1.109	14.6	423.2
S3	5.3	− 41.3	0.866	18.5	493.5
S4	6.2	− 39.2	0.697	31.6	527.1
S4F1	6.5	− 38.0	0.577	34.8	1586.6
S4F2	8.1	− 37.6	0.571	35.8	2402.6
S4F3	9.4	− 36.4	0.550	37.3	1234.4

**Figure 9:**
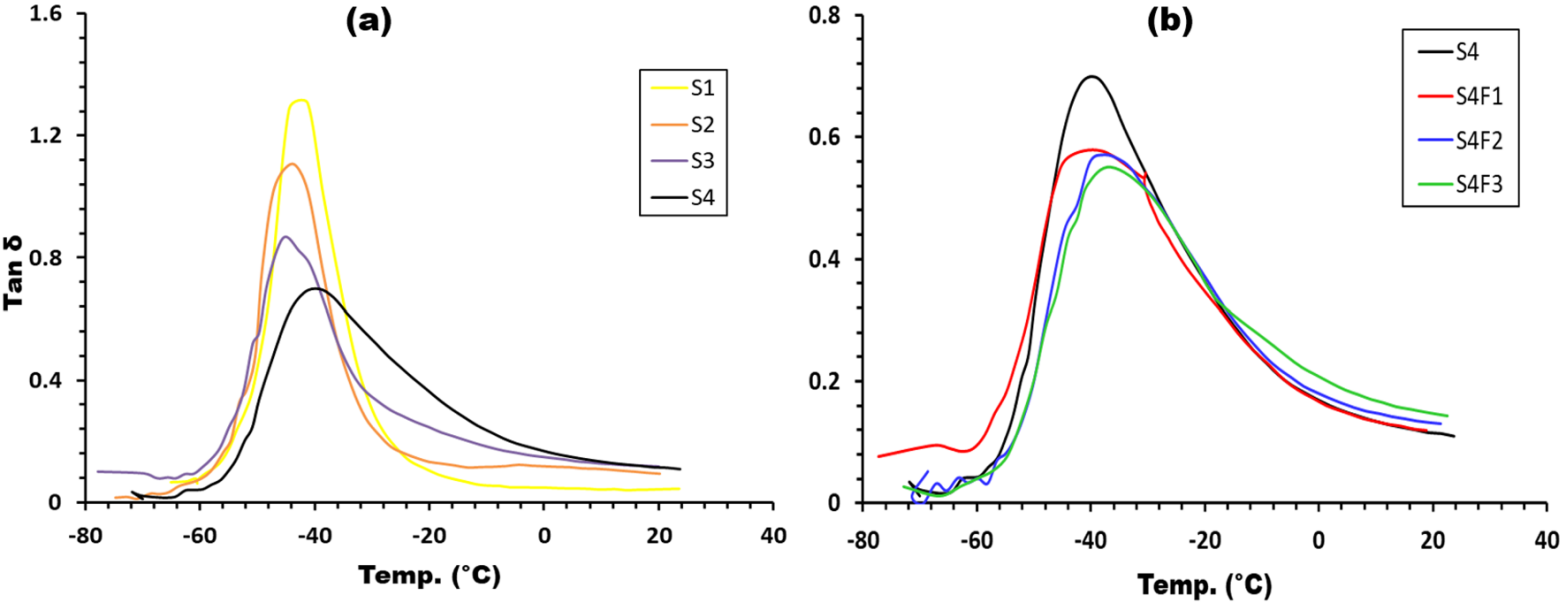
exhibits Tan δ vs. temperature for samples with (**a**) different waste rubber concentrations and (**b**) Fe_2_O_3_ concentrations.

In Fig. 9a,b. the impact of temperature on damping parameters (tan δ) is elucidated. The glass transition temperature (T_g_), pinpointed as the peak point of the damping parameter, signifies the initiation of chain segment motion, including Brownian motion and stress relaxation^33^. The transition region, characterized by robust damping, results from the cooperative motion of a significant number of chain segments under deformation. This cooperative motion leads to maximal damping, and the T_g_, height, and full-width half maximum (FWHM) of tan δ peaks offer valuable insights into the structure and properties of polymer composites^34,35^. These parameters are influenced by filler concentration, type, distribution, and filler-matrix interaction, as comprehensively detailed in Table 4.

The glass transition temperature (T_g_) shifted as the waste rubber and Fe_2_O_3_ concentrations were varied. The T_g_ values ranged from − 45.5 °C for pure EPDM (S1) to − 43.9 °C, − 41.3 °C, and − 39.2 °C for samples S2, S3, and S4, respectively. The addition of Fe_2_O_3_ to sample S4 resulted in T_g_ values of − 38 °C, − 37.6 °C, and − 36.4 °C for samples S4F1, S4F2, and S4F3, respectively. The height of the damping peaks decreased while the FWHM increased for all prepared samples with varying waste rubber and ferric oxide concentrations. These observations indicate good interfacial adhesion and efficient stress transfer mechanisms.

**Figure 10:**
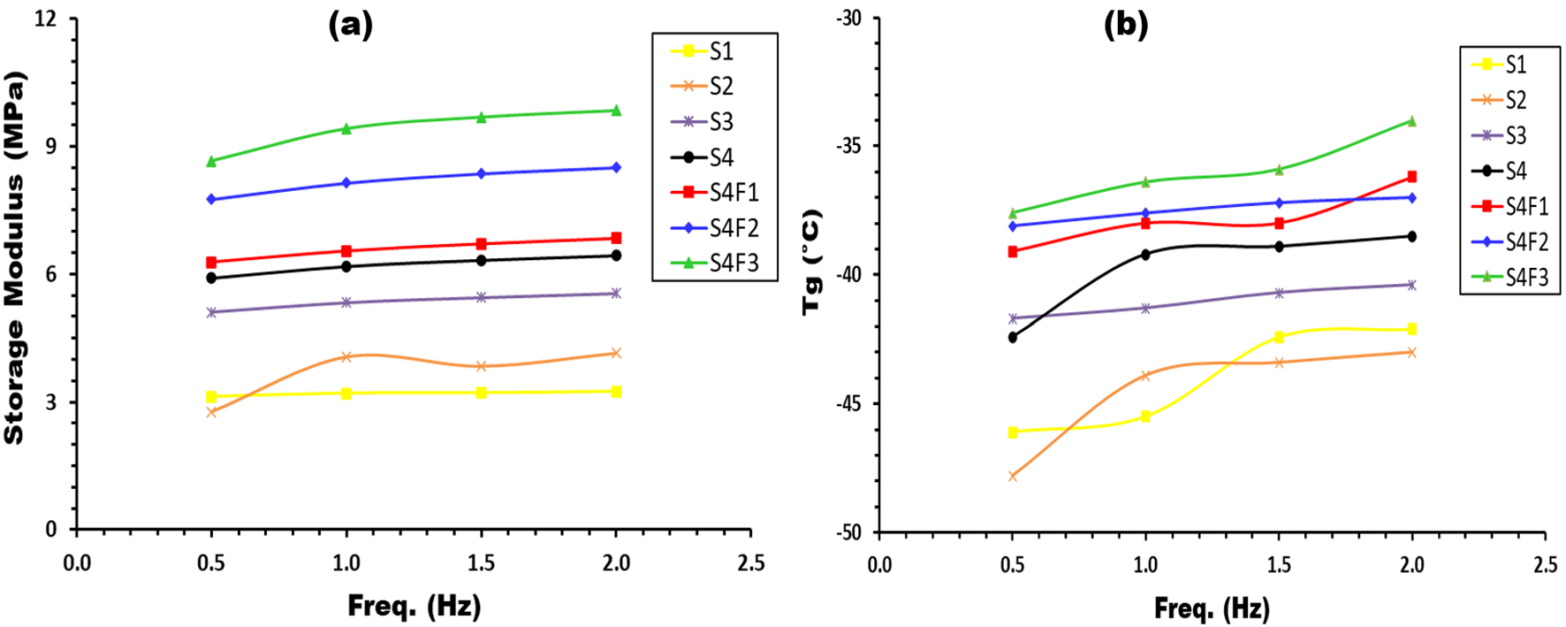
Frequency dependency of (**a**) storage modulus and (**b**) glass transition temperature of samples with different waste rubber concentrations and Fe_2_O_3_ concentrations.

The interplay between storage modulus and frequency at room temperature is investigated in Fig. 10a for samples with varying waste rubber and ferric oxide concentrations. A noticeable increase in storage modulus is observed with rising frequency, attributed to molecular rearrangement under continuous stress. Higher frequency measurements yield larger storage modulus values, while lower frequency measurements result in lower values^36,37^. This behavior is explicable by the shorter time required for molecular chain oscillation at high frequencies compared to the time needed for relaxation. Continuous oscillatory forces during long-time measurements reveal high elasticity, contributing to an increase in the storage modulus value^37^.

Additionally, Fig. 10b illustrates the correlation between glass transition temperature (T_g_) and frequency for all prepared samples with differing waste rubber and ferric oxide concentrations. T_g_ increases with higher concentrations of waste rubber and Fe_2_O_3_, reducing the material's flexibility and constraining the mobility of polymeric molecule segments at relaxation temperatures. This observation affirms the reinforcing efficacy of these components. Furthermore, T_g_ increases with frequency, a relationship explicable by the Arrhenius relationship, enabling the determination of activation energy (E_a_) for the relaxation process at the transition area^33^. The values of activation energy (E_a_) for all samples, detailed in Table 4, increase with waste rubber and Fe_2_O_3_ concentrations, signifying a decrease in polymer chain mobility and a consequent requirement of higher activation energy for chain motion. These findings contribute to a nuanced understanding of the dynamic mechanical response of polymer composites to varying temperature conditions.

### Radiation shielding parameters

In the pursuit of enhancing radiation shielding efficiency, this study employs a gamma-ray spectrometer system to investigate the linear attenuation coefficient (μ) values at 662keV. The research extends to the calculation of mass attenuation coefficients (μm), half-value layer (HVL), tenth-value layer (TVL), relaxation length *λ* and density for EPDM/waste rubber composites with varying concentrations of Fe_2_O_3_ filler (0, 50, 100, 150 phr). Table 5 presents the results, emphasizing the role of the mass attenuation coefficient as a crucial parameter independent of absorber density and physical state) of the absorber^12^.

**Table 5:** The experimental linear attenuation coefficients (μ) and important parameters for shielding composites.

	At gamma energy 662 keV					
Samples	μ (cm^−1^)	μ_m_(cm^2^/g)	HVL (cm)	TVL (cm)	Density (g/cm^3^)	λ (cm)
S4	0.1235	0.1317	5.612	18.644	0.9375	8.097
S4F1	0.1282	0.1146	5.406	17.960	1.1179	7.800
S4F2	0.1556	0.1269	4.454	14.798	1.226	6.426
S4F3	0.1947	0.1560	3.560	11.826	1.248	5.136

The findings reveal a direct correlation between sample thickness, Fe_2_O_3_ filler concentration, and the gradual increase in both linear and mass attenuation coefficients. Concurrently, composite density rises with higher filler content. The observed decrease in HVL and TVL with escalating attenuation coefficients underscores the shielding capability of the composite polymer against γ-radiation at 662 keV or below.

Significantly, sample S4F3 exhibits the highest µ values at 662 keV, attributable to its relatively elevated Fe_2_O_3_ concentration. Linear attenuation, contingent upon concentration, density of the filler in the matrix and the bulk material as well as, and incident photon energy. Table 5 demonstrates a notable 36.57% decrease in HVL and TVL values for S4F3 compared to S4, reinforcing the material's enhanced shielding properties. The relaxation length (λ), reciprocally of the linear attenuation coefficient (μ), emerges as a pivotal parameter in gauging shielding efficiency. Lowest λ values (S4F3) indicate superior shielding properties at 662 keV, with the composites exhibiting favorable characteristics. Notably, materials with shorter relaxation lengths are more efficient at absorbing low-energy photons over shorter distances, while high-energy photons require longer distances for energy dissipation^38^.

**Figure 11:**
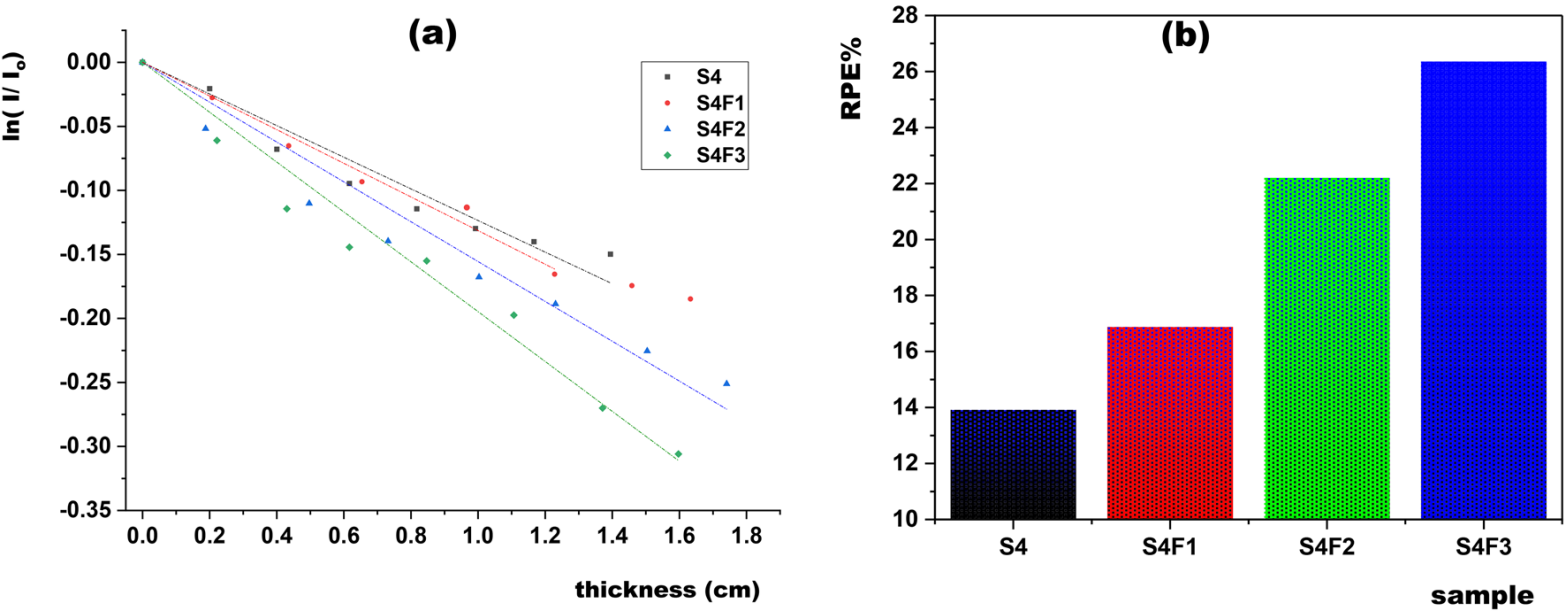
(**a**) ln I/Io versus the thickness for EPDM/waste rubber loaded with different concentration of Fe_2_O_3_, (**b**) Radiation protection efficiency (RPE%) for different concentrations of Fe_2_O_3_.

Figure 11a elucidates the thickness-ln(I/Io) relationship, facilitating μ calculation. Concurrently, Fig. 11b presents radiation protection efficiency (RPE%) values, ranging from 13.91% (unfilled S4) to 26.35% (S4F3) at 662keV, demonstrating the effectiveness of Fe_2_O_3_ concentration in enhancing shielding capabilities.

A comprehensive comparison in Table 6 assesses the shielding parameters of S4F3 against various materials. The results affirm the superior shielding efficiency of the studied composite compared to some established shielding materials.

**Table 6:** Evaluation of many shielding parameters for various specimens with the present S3 examined sample at gamma energy 662 keV.

Samples	HVL (Cm)	TVL (Cm)	µ_m_ (Cm^2^/g)	λ (Cm)	References
SBR5/Pb_3_O_4_	1.35	4.48	0.0930	2.78	^15^
Bismuth Borate Glass	2.38	7.91	0.083	3.43	^39^
Polyvinyl alcohol (PVA)	7.70	25.5	0.082	11.1	^40^
PVC-H30	3.42	11.36	0.0795	4.93	^41^
PVA/Fe_3_O_4_ NP_S_	1	11.91	0.382	1.44	^39^
WR/EPDM/Fe_2_O_3_ (S4F3)	3.560	11.826	0.1560	5.14	Present study
Borated polyethylene	7.10	23.55	0.0821	11.17	^42^
Ordinary concrete	7.74	25.70	0.0814	11.16	^43^

In conclusion, this investigation establishes the radiation shielding prowess of EPDM/waste rubber composites with Fe_2_O_3_ filler, providing valuable insights into the interplay of filler concentration, composite characteristics, and shielding effectiveness at 662keV.

## Conclusion

By effectively managing waste, individuals may have a significant impact on both society and the global community. While it may not be possible to totally eliminate waste, adopting environmentally friendly practices that focus on waste reduction and reuse is always an option. Through the preparation of a waste rubber/EPDM composite, we conducted an investigation which revealed that the specific gravity results confirm the accurate preparation of the samples. The water absorption results indicated that all samples fall within the permissible range for use in building applications. ATR-FTIR measurements showed the existence of C–H stretching, symmetric and asymmetric vibration also vibrations of dienes are observed and the bending vibration of Fe–O bond. The thermal stability of EPDM rubber was significantly enhanced by including waste rubber, as seen by a shift in thermal decomposition. Conversely, the inclusion of Fe_2_O_3_ resulted in just a slight improvement in thermal stability. The dynamic mechanical characteristics show a notable enhancement as the concentrations of waste rubber and ferric oxide grow. The investigation focused on assessing the shielding effectiveness of different EPDM/WR-Fe_2_O_3_ composites by measuring parameters such as linear attenuation coefficient (μ), mass attenuation coefficient (μ_m_), half-value layer (HVL), tenth-value layer (TVL), radiation protection efficiency (RPE %), and relaxation length for gamma radiation. The composites offer sufficient shielding capabilities and are valuable in various shielding applications, particularly in medical fields where low to intermediate energy is utilized This lightweight material can be utilized as a wall lining to decrease the quantity of lead bricks or sheets required for shielding, thereby reducing the emission of backscattered radiation in nuclear medicine departments and X-ray diagnostic rooms.

## Data Availability

The data that support the findings of this study are available from the corresponding author upon reasonable request.
